# Supplementary material for: Antioxidants and azd0156 Rescue Inflammatory Response in Autophagy-Impaired Macrophages
Source: Int J Mol Sci. 2023 Dec 21;25(1):169. doi: 10.3390/ijms25010169 (PMC10779076; doi:10.3390/ijms25010169)
Supplement: Supplementary file 1 [file ijms-25-00169-s001.zip › ijms-2746333-supplementary.pdf]

Supplementary Table S1_qPCR primers of Human Genes

Primer Name	Forward Sequence	Reverse Sequence:
SQSTM1	TGTGTAGCGTCTGCGAGGGAAA	AGTGTCCGTGTTTCACCTTCCG
INOS	GCTCTACACCTCCAATGTGACC	CTGCCGAGATTTGAGCCTCATG
ARG1	TCATCTGGGTGGATGCTCACAC	GAGAATCCTGGCACATCGGGAA
IL1B	CCACAGACCTTCCAGGAGAATG	GTGCAGTTCAGTGATCGTACAGG
CXCL2	GGCAGAAAGCTTGTCTCAACCC	CTCCTTCAGGAACAGCCACCAA
CXCL3	TTCACCTCAAGAACATCCAAAGTG	TTCTTCCCATTCTTGAGTGTGGC
MT2A	GAGTGCAAATGCACTTCGTGCAA	GCGTTCTTTACATCTGGGAGCG
VEGFA	TTGCCTTGCTGCTCTACCTCCA	GATGGCAGTAGCTGCGCTGATA
SOD2	CTGGACAAACCTCAGCCCTAAC	AACCTGAGCCTTGGACACCAAC
IL2	AGAACTCAAACCTCTGGAGGAAG	GCTGTCTCATCAGCATATTCACAC
IL8	GAGAGTGATTGAGAGTGGACCAC	CACAACCCTCTGCACCCAGTTT
CCL3	ACTTTGAGACGAGCAGCCAGTG	TTTCTGGACCCACTCCTCACTG
CCL4	GCTTCCTCGCAACTTTGTGGTAG	GGTCATACACGTACTCCTGGAC
H3F3A	ACAAAAGCCGCTCGCAAGAGTG	TTTCTCGCACCAGACGCTGGAA
THBS1	GCTGGAAATGTGGTGCTTGTCC	CTCCATTGTGGTTGAAGCAGGC
ATM	TGTTCCAGGACACGAAGGGAGA	CAGGGTTCTCAGCACTATGGGA
ATR	GGAGATTTCCTGAGCATGTTCGG	GGCTTCTTTACTCCAGACCAATC
H2AX	CGGCAGTGCTGGAGTACCTCA	AGCTCCTCGTCGTTGCGGATG
RAD23	GCGTTACTACAGCAGATAGGTCG	CAGCTTCTGCAATTCCTCCACTG
RAD50	GGAAGAGCAGTTGTCCAGTTACG	GAGTAAACTGCTGTGGCTCCAG
UNG	CCACACCAAGTCTTCACCTGGA	CCGTGAGCTTGATTAGGTCCATG
DDB1	CATTCCTCGCTCCATCCTGATG	CCTTCTTACGGTCGCTCAACAG
ACTB	CACCATTGGCAATGAGCGGTTC	AGGTCTTTGCGGATGTCCACGT
GADPH	GTCTCCTCTGACTTCAACAGCG	ACCACCCTGTTGCTGTAGCCAA

Supplementary Table S2_qPCR primers of Mouse genes

Primer Name	Forward Sequence	Reverse Sequence:
Cd45	CTTCAGTGGTCCCATTGTGGTG	TCAGACACCTCTGTCGCCTTAG
Mmp2	CAAGGATGGACTCCTGGCACAT	TACTCGCCATCAGCGTTCCCAT
Mmp9	GCTGACTACGATAAGGACGGCA	TAGTGGTGCAGGCAGAGTAGGA
Cxcl2	CATCCAGAGCTTGAGTGTGACG	GGCTTCAGGGTCAAGGCAAACT
Ccl4	ACCCTCCCACTTCCTGCTGTTT	CTGTCTGCCTCTTTTGGTCAGG
Thbs1	GGTAGCTGGAAATGTGGTGCGT	GCACCGATGTTCTCCGTTGTGA
Ung	ATGGACCCAGATGTGCGACATC	CTGGTCTTTGGACACTGAAGCAG
Ddb1	GTAGCCACTACCTCCTTTGTGC	GAGAACGGAATGTCCTCAACACC
Actb	CATTGCTGACAGGATGCAGAAGG	TGCTGGAAGGTGGACAGTGAGG
Gadph	CATCACTGCCACCCAGAAGACTG	ATGCCAGTGAGCTTCCCGTTCAG
